# Cancer pain treatment during the COVID-19 pandemic: institutional recommendations

**DOI:** 10.6061/clinics/2020/e2208

**Published:** 2020-08-14

**Authors:** Angela Maria Sousa, Thiago Ramos Grigio, Hazem Adel Ashmawi, Ulysses Ribeiro Júnior

**Affiliations:** I Anestesia, Hospital das Clinicas HCFMUSP, Faculdade de Medicina, Universidade de Sao Paulo, Sao Paulo, SP, BR.; II Instituto do Cancer do Estado de Sao Paulo (ICESP), Hospital das Clinicas HCFMUSP, Faculdade de Medicina, Universidade de Sao Paulo, Sao Paulo, SP, BR.; III Anestesiologia Experimental LIM-08, Hospital das Clinicas HCFMUSP, Faculdade de Medicina, Universidade de Sao Paulo, Sao Paulo, SP, BR.

Pain is one of the most frequent and feared symptoms in patients with cancer. A recent metanalysis (1) revealed a pain prevalence of 39.3% after curative treatment, 55.0% during anticancer treatment, and 66.4% in advanced disease patients (1). Among cancer patients, 38.0% reported moderate to severe pain (1,2), causing functional status impairment and poor quality of life. Despite the marked improvement in the treatment of cancer (2), 30% of patients still develop refractory pain, requiring invasive procedures to achieve partial or complete relief (3).

Interventional pain procedures can be classified into neuromodulation and neuroablative. Neuromodulation is the functional interruption of pain pathways by intraspinal administration of drugs (4). Epidural infusion of opioids plus local anesthetics is a common example of neuromodulation in the postoperative period. In contrast, neuroablative procedures are used to treat chronic cancer pain and consist of physical interruption of pain pathways by surgical, chemical, or thermal means (4). Ablative procedures promote better pain control and quality of life than pharmacological treatment, but they require hospitalization and are performed with the use of fluoroscopy, computerized tomography, and/or ultrasound.

The COVID-19 pandemic forced the pain specialists and institutions to balance the risks of infection versus the benefits of pain procedures (5,6). Thus, the purpose of this recommendation is to establish institutional routines that may reduce the risk of contamination of patients and health professionals during the COVID-19 pandemic, in the event of performing invasive pain procedures.

## General Recommendations

1. Outpatient appointments: full consideration should be given to minimize patients congregating in a waiting room. The use of telemedicine is recommended for follow-up of outpatients (5,6).
2. In-hospital visits: to preserve health resources and protective equipment, it is considered essential to reduce the number of people examining the patients. Specialists’ consultations should be limited to the essential. When the interventional procedure is indicated, the pain specialist should examine the patient (5,6).
3. During clinical evaluation, the pain specialist should use surgical masks and gloves. For high-risk patients, professionals should protect themselves by wearing particulate-filtering respirators (N-95).
4. Individuals at high risk of COVID-19 infection should be tested before hospitalization (7) within three to five days before the procedure. Currently, with community dissemination, all cases can be suspected positive for COVID-19, even asymptomatic patients.
5. Unlike patients with non-oncological pain (5,6), interventional pain procedures can never be postponed. Cancer patients are usually at risk of worsening their clinical condition and sometimes such procedures are the best option to improve their quality of life.
6. During the interventional procedure, it is recommended that individuals ought to have full protective garments, including an N95 mask, ocular protection, and double gloves. Ensure that patients wear a surgical mask besides usual surgical gowns. Further, ensure that the fluoroscopy and ultrasound devices have protective covers. Additionally, reduce the number of people present during the procedure (5,6). A negative pressure operation room should be utilized for performing the procedure.
7. Pain procedures can be classified (5,6) as
  - Urgent Procedures: intrathecal pump refill or malfunctioning of neurostimulators; intrathecal catheter infection.
  - Semi-urgent procedures: refractory cancer pain; patients hospitalized due to pain; suspected opioid abuse.
8. If there is a need for hospitalization for an outpatient procedure, RT-PCR for SARS CoV2 and chest tomography should be performed (7). The patient should be kept hospitalized for the shortest duration as possible.

## Therapeutic recommendations:

1. Corticosteroids: Steroids can suppress the immune system and are related to infections, including pneumonia (8). The injection of intra-articular steroids is associated with an increased risk for influenza infection (9).
  Following lumbar facet joint injections, cortisol levels are suppressed for an average of 4.4 days (10). Although COVID-19 induces an exaggerated immune response, steroids are only recommended for refractory shock (11). One should consider the risk/benefit of steroid injections and reduce the dose, especially in high-risk patients during the current COVID-19 pandemic (5,6).
2. Non-steroidal anti-inflammatory drugs (NSAIDs): At the beginning of the spread of the SARS-CoV-2 infection, European doctors indicated the non-use of ibuprofen or another NSAID, due to the risk of increasing levels of angiotensin-converting enzyme (ACE); thus, worsening COVID-19 (12). However, these results have not been proven. It should be noted that NSAIDs can mask some early symptoms of the disease, such as fever and myalgia (5).
3. Assess the risk/benefit of administering opioids. Opioids act on the hypothalamic/pituitary/adrenal (HHA) axis and activate the sympathetic nervous system (SNS). The SNS innervates lymphoid organs, such as the spleen, and this activation induces the release of biological amines that suppress the proliferation of splenic lymphocytes and the cytotoxicity of NK cells (13). Additionally, the prolonged use of opioids increases the activity of HHA and the production of glucocorticoids, which also decreases the cytotoxicity of NK cells (14). On the other hand, pain itself is immunosuppressive, and not prescribing opioids for the possibility of immunosuppression can be even more devastating.
4. Interventional procedures reduce opioid consumption and improve the quality of analgesia (4). However, most invasive procedures for cancer patients are performed in the inpatient regimen, which would increase their exposure to infection. The best option is to use common sense and evaluate case by case, especially during board discussions.

Cancer patients are immunocompromised and more susceptible to infections than the general population. These patients are older, have higher angiotensin-converting enzyme-2 (ACE2) expression, and more comorbidities (15). They are at higher risk of adverse outcomes (16), including intensive care admission, a requirement for mechanical ventilation, or death (16). Moreover, these patients are twice more likely to be diagnosed with COVID-19 than the general population (17,18).

A pragmatic approach is required when deciding whether to offer interventional therapies for treating cancer pain. The potential benefits and possible risks need to be considered in a scenario where social isolation and confinement at home are guidelines established by global health entities (19). Neuroablation can provide long-term pain control and should be considered for treating severe cancer pain (3).

Therefore, the implementation and optimization of the pain control protocol described above would intervene positively in the quality of life of our patients, minimizing the risks during the COVID-19 pandemic.
